# Maternal urinary 2-hydroxynaphthalene and birth outcomes in Taiyuan, China

**DOI:** 10.1186/s12940-018-0436-4

**Published:** 2018-12-20

**Authors:** Jisheng Nie, Jinyu Li, Lin Cheng, Yanning Li, Yunjun Deng, Zhiwei Yan, Lei Duan, Qiao Niu, Frederica Perera, Deliang Tang

**Affiliations:** 1 Department of Occupational and Environmental Health, School of Public Health, Shanxi Medical University, Xinjiannan Road 56, Taiyuan, 030001 China; 2 Department of Environmental Health Sciences, Mailman School of Public Health, Columbia University, 722W. 168th Street, New York, NY 10032 USA

**Keywords:** Naphthalene, 2-hydroxynaphthalene, Birth outcomes, Polycyclic aromatic hydrocarbons, Prenatal exposure

## Abstract

**Background:**

Naphthalene is the simplest polycyclic aromatic hydrocarbon (PAH). It is easily emitted into the atmosphere, posing a significant risk to human health. However, limited studies have described the impact of naphthalene exposure on birth outcomes. In this study, we investigated the association between the maternal urinary metabolites of naphthalene, 2-hydroxynaphthalene (2-OH NAP), and birth outcomes.

**Method:**

In the present study, four urinary PAH metabolites were measured in 263 pregnant women during late pregnancy. Multiple linear regression analysis was used to analyze the relationship between the concentrations of 2-OH NAP and birth outcomes, and restricted cubic spline models were further used to examine the shapes of the dose-response association.

**Result:**

General linear models showed that prenatal urinary 2-OH NAP was associated with lower birth weight (BW) (− 4.38% for the high vs. low exposure group of 2-OH NAP; p for trend = 0.049) and higher cephalization index (CI) (4.30% for the high vs. low exposure group of 2-OH NAP; p for trend = 0.038). These associations were linear and significant when 2-OH NAP was modeled as a continuous variable in restricted cubic spline models (P _linear_ = 0.0293 for 2-OH NAP and BW; P _linear_ = 0.0326 for 2-OH NAP and CI). Multiple linear regression data indicated that each 1 ln-unit increase in 2-OH NAP was significantly associated with a 2.09 g/cm increase in the CI. The associations among 2-OH NAP, BW, and CI were also observed in a subset of participants residing close to arterial traffic.

**Conclusion:**

Our data indicated that prenatal exposure to naphthalene had an adverse effect on fetal birth outcomes, especially the brain development index. Reduced exposure to naphthalene may improve newborn health outcomes. In Taiyuan, naphthalene may result from traffic pollution.

**Electronic supplementary material:**

The online version of this article (10.1186/s12940-018-0436-4) contains supplementary material, which is available to authorized users.

## Background

Naphthalene, the simplest polycyclic aromatic hydrocarbon (PAH), is a natural constituent of petroleum and a product of incomplete combustion of organic materials and petroleum products. Naphthalene is detected in cigarette smoke, in automobile exhaust and during combustion of petroleum products [1]. It is easily emitted into the atmosphere in gaseous form and contributes to air pollution. Most studies address the effect of five- to six- ring PAHs on human health due to the contaminants related to genotoxicity, carcinogenic capability, and the specificity of chemical structure. The effects of naphthalene on human health are often overlooked because of the diminished toxicity detected in a lower dose range for this PAH.

Studies demonstrate that exposure to naphthalene can destroy red blood cells and cause confusion, nausea, vomiting, and jaundice [2, 3]. The US national toxicology program notes that naphthalene exposure can increase the incidence of cancer [4]. Another study also suggests that naphthalene levels should be thoroughly monitored in industrial cities with high levels of PAH [5]. In China, the use of naphthalene in mothballs is forbidden due to its potential toxicity on human health [6]. The above information suggests that the toxicity of naphthalene to human health should not be overlooked. The fetus is more susceptible to toxicological consequences of environmental toxicants because of its physiologic immaturity, weak capability to detoxify toxic chemicals, and deficient immune system responses [7, 8]. Recent studies have also revealed the presence of naphthalene in placental tissue and indicated that naphthalene can easily cross the placental barrier [9, 10]. Therefore, the impact of prenatal naphthalene exposure on offspring health should be focused.

Birth outcomes are objective and visual indicators that can reveal the nutritional and health status of infants and fetuses and predict the possibility and risks of adulthood diseases [11–15]. This association of birth outcomes with PAH metabolites has been reported recently. Polanska et al. [16] detected a link between the sum of hydroxyphenanthrene levels and the higher cephalization index (CI). The relationship between 1-hydroxypyrene (1-OH PYR) and birth outcomes has also been investigated in previous studies [9, 17, 18]. However, the association between naphthalene exposure and birth outcomes has rarely been tested in epidemiological studies. In the in vivo experiment, pregnant rats administered oral naphthalene had lower weights and fewer pups per litter than control rats [19, 20]. Taiyuan, China, is an industrialized city with relatively high levels of PAH in air pollution attributed to multiple sources, especially coal burning for industrial purposes and automobile exhaust [21]. The emission load of naphthalene is also the highest among 16 PAHs identified by the US EPA [22]. Therefore, using the Taiyuan Mother and Child Cohort Study data, we aimed to explore the association between 2-OH NAP and birth outcomes in newborns and provide guidance for reducing exposure to hazardous pollutants.

## Subjects and methods

### Study population

Pregnant women who waited for delivery during the third trimester of pregnancy (≥ 35 weeks) in the Sixth Hospital of Shanxi Medical University and The Eighth People’s Hospital of Taiyuan were invited to participate in the Taiyuan Mother and Child Cohort Study. Pregnant women who had resided in Taiyuan for at least 1 year, were nonsmokers, were ≥ 18 years of age, and had a single gestational viable fetus were considered eligible for participation in the cohort study. A total of 287 pregnant women were included in the study. According to self-reported and medical record data, 17 subjects who had a chronic disease were excluded from the study. Urine samples and cord blood were collected for analysis from the 263 pregnant women included in the analysis. Prior to enrolling subjects, all participants gave written informed consent and the study was approved by the Research Ethics Committee of Shanxi Medical University.

### Personal interview questionnaire

Immediately following consent to participate, participants were asked to complete a validated questionnaire supervised by a trained interviewer during the perinatal period. The questionnaire collected maternal demographic information (place of residence, duration of residence, age, health condition, education, occupation, and socioeconomic status), characteristics of the newborns (birth outcomes, vital signs and condition during delivery), and maternal individual behaviors and lifestyles (active and passive smoking, frequency of eating fried, broiled and barbecued meat and consumption of alcohol during the whole period of pregnancy). Passive smoking refers to self-reported exposure to tobacco smoke more than 15 min every day [23]. The definition of arterial road was consistent with that in the *code for transport planning on urban road*, which includes 40–60 km/h speed, more than 4 automobile lanes, 3.5-m lane width, and a separation zone [24]. The determination of 35 m as a cutoff point was based on a previous study [25]. Within a 35- m distance from the residence to the arterial road, the level of heavy metals in the roadside soil gradually increases with increasing distance, and beyond 35 m, the level of heavy metal is relatively stable. Spouse demographic information was also collected. To ensure the quality of the questionnaire, personal interview time was no less than 45 minutes.

### Biological sample collection and analysis

Urine samples (30–50 mL) were collected from the pregnant women during the last antenatal physical examination. The urinary samples were transported to the laboratory immediately, repackaged into 5-mL cryogenic vials, and then stored at − 80 °C until chemical analyses. Umbilical cord blood was drawn immediately after delivery into whole blood tubes with spray-coated EDTA (BD, Franklin Lakes, NJ, USA) and then repackaged into 1-mL cryogenic vials and stored at − 80 °C until chemical analyses.

The PAH metabolites measured in the present study included 2-hydroxynaphthalene (2-OH NAP), 2-hydroxyfluene (2-OH FLU), 9-hydroxyphenanthrene (9-OH PHE), and 1-hydroxypyrene (1-OH PYR). The four PAH metabolites were measured using high-performance liquid chromatography with a fluorescence detector (HPLC-FLD (Shimadzu, Kyoto, Japan)) as described previously [26, 27]. Briefly, 5 mL of thawed urine sample was hydrolyzed with 25 μL β-glucuronidase, loaded onto a Sep-Pak C_18_ (6 cc, 500 mg) cartridge (Waters, Mil-ford, MA, USA) and condensed by dry nitrogen purge to obtain a 0.5-mL extract. The extract was then analyzed using HPLC-FLD to determine 2-OH NAP, 2-OH FLU, 9-OH PHE and 1-OH PYR levels. The details of the separation condition and program were as follows. Four PAH metabolites were separated using a C_18_ column, an oven temperature of 35 °C, manual injection of 20 μL and flow of 1.0 mL/min, with a gradient elution program using methanol and water (methanol: 60% during the first 10 min, increasing to 70% in the following 15 min to 30 min, then returning to 60% after 35 min). The wavelength excitation (Ex) and emission (Em) of the fluorescence detector were as follows: (1) 2-OH NAP (227/355), (2) 2-OH FLU (275/330), (3) 9-OH PHE (255/385), and (4) 1-OH PYR (242/385). The calibration standards of 2-OH NAP, 2-OH FLU, 9-OH PHE and 1-OH PYR were purchased from Sigma-Aldrich Co. (USA). β-Glucuronidase (glucuronidase activity ≥100,000 units/mL) was obtained from Roche Co. (Germany); sodium acetate and methanol (HPLC Grade) were obtained from Sigma-Aldrich Co. (USA) and Fisher Scientific Co. (USA), respectively. The limit of detection (LOD) was determined as the lowest calibration standard at which analytes provided a signal-to-noise ratio (S/N) of 3. The linearity (expressed as R^2^), LOD, precision (expressed as relative standard deviation (RSD)), and mean recovery rate were 0.9998–1, 0.01–0.05 μg/L, 0.17–2.4%, and 83.75–100.50%, respectively. Reagent blanks and urine samples were analyzed, and none of the PAH metabolites were detected in the blanks, indicating that the experimental process did not introduce contamination. To control for urine dilution, we adjusted PAH metabolite concentrations for urine creatinine levels, which were detected using alkaline picrate [26, 27] and quantified by spectrophotometry (SpectraMAx M2, Molecular Devices, USA) at a wavelength of 520 nm.

Urinary phenol was measured using a Gas Chromatographic with Hydrogen Flame Ionization detector (GC, Shimadzu, Kyoto, Japan) according to Health Industry Standards of the People’s Republic of China WS/T 50–1996 [28]. Briefly, the urine sample was heated with hydrochloric acid to hydrolyze the phenol and extracted with diethyl ether. Urinary phenol was separated from the normal human metabolite p-cresol by an FFAP column and was detected by a Hydrogen Flame Ionization detector. Then, according to the peak height, we used the external standard method to quantify the urinary phenol levels.

The cord blood lead measurement was performed with a PinAAcle900Z atomizer absorption spectrophotometer coupled to a THGA graphite furnace and a programmable sample dispenser (PerkinElmer Company, MA, USA), as described previously [29]. The LOD was 0.1 μg/L. All of the cord blood lead levels in our study were above the LOD. Regular quality control procedures included instrument calibration, procedural blanks, replicates, and certified reference materials to ensure the accuracy of measurement. The standard reference materials (Contox; Kaulson Laboratories, Inc., NJ, USA) were used in the daily calibration. All biological samples were analyzed by Shanxi Medical University.

### Anthropometric measurement of the newborn

We summarized information on birth outcomes from maternal and newborn medical records. The anthropometric indicators of the newborn in the present analyses included birth weight (BW) (in grams), birth length (BL), birth head circumference (BHC) (in centimeters), and two growth proportionality indices: ponderal index (PI) and CI. The PI [BW (g) / (BL (cm)) ^3^ × 10^2^] is similar to body mass index and is most commonly used in pediatrics [30]. The CI [BHC (cm) / BW (g) × 10^4^] is a validated predictive indicator of intrauterine growth retardation that affects fetal brain development [31]. A higher CI reflects a greater degree of brain vulnerability and more severe clinical pathology as well as the likelihood of cerebral palsy and severe psychomotor retardation [32].

### Statistical analysis

The collected data were analyzed using SPSS 16.0 (SPSS Inc., Chicago, IL, USA) and SAS 9.4 (SAS Institute, Inc., Cary, NC). A bilateral *p*-value less than 0.05 was considered statistically significant. Continuous variables are expressed as the mean ± standard deviation (SD) or median and interquartile range (IQR). Categorical variables are presented as numbers and frequencies (%). Natural log transformation was applied to all the exposure metrics (2-OH NAP, 2-OH FLU, 9-OH PHE, and 1-OH PYR) to correct skewed distributions. The concentration of each PAH metabolite below the LOD was assigned a value corresponding to one-half the LOD. Spearman correlation analysis was used to evaluate the association between PAH metabolites and birth outcomes. General linear models and restrict cubic spline models were used to explore the dose-response association between PAH metabolites and birth outcomes after adjusting for potential confounders. Multiple linear regressions were used to estimate the effect of PAH exposure on birth outcomes. To assess the presence of collinearity and independence among the covariates, we ran the variance inflation factor (VIF), tolerance, and Durbin-Watson (DW) test on all linear regression models [9, 33–35]. The VIF value of each variable was far less than 10, the tolerance value of each variable was more than 0.1, and the DW value also met the requirements, indicating that there was no strong collinearity problem in any independent variable (Additional file 1: Table S1 and Table S2). Tikhonov regularization was also used to eliminate the effect of multicollinearity on regression models and to validate the association between PAH metabolites and birth outcomes. Power analysis was used to evaluate the power of sample size. Potential confounding factors [maternal age, maternal BMI, parity, gender of newborn, gestational age, eating grilled meat, passive smoking] were included in the models based on previous literature. Considering that benzene and lead were also the main components of traffic-related pollutants, we further adjusted for urinary phenol and cord blood lead levels in regression models.

## Results

Table 1 shows the baseline characteristics of 263 mother-newborn pairs analyzed in the present study. The age of pregnant women ranged from 19 to 40. More than half of pregnant women were passive smokers (57.4%) and reported their residence adjoining arterial traffic (< 35 m) (57.0%). The number of female babies was lower than the number of male babies in the population (44.9 and 55.1%, respectively). Most newborns were registered as a first child (72.6%). The BW, BL, BHC, PI and CI of infants at the time of delivery were not significantly different between females and males. Table 2 demonstrates the urinary concentration distribution of four PAH metabolites. To compare with similar studies, we show the creatinine-corrected concentrations in this table. The median and interquartile range (IQR) of PAH metabolites, including 2-OH NAP, 2-OH FLU, 9-OH PHE, 1-OH PYR and ΣOH-PAH, in maternal urine were 6.34 (4.03–9.41), 3.47 (2.23–5.40), 2.88 (1.46–4.68), 1.83 (0.90–3.03), and 14.81 (10.40–22.07) μg/g Cr, respectively. The median value of 2-OH NAP was the highest (6.34 μg/g Cr) in this study. There were no direct data in our study about atmospheric PAH levels in Taiyuan. Jing JQ et al. [22] showed that the highest emission load was naphthalene in Taiyuan among 16 PAHs identified by the US EPA, with a 39.18% contribution rate (Additional file 1: Table S3). The PAH levels in the emission load were consistent with maternal urinary PAH metabolite levels. There was a seasonal difference with respect to PAH metabolite levels in maternal urine. The 2-OH NAP level is relatively high in January and February, while the other three PAH metabolites were basically unchanged (Additional file 1: Figure S1). Spearman correlation analysis showed that there were positive correlations among PAH metabolites. Maternal urinary 2-OH NAP was significantly associated with BW (*r* = − 0.15) and the PI (*r* = − 0.13) with *P*-values of < 0.05 for both. A similar pattern was observed for 2-OH FLU with an r (*P*-values) of − 0.15 (< 0.05) and − 0.18 (< 0.05) for BW and the PI, respectively. In contrast, maternal urinary 2-OH NAP and 2-OH FLU showed significant positive relationships with the CI (*r* = 0.17; *r* = 0.15), with P-values < 0.05 for both. The 9-OH PHE and 1-OH PRY levels were inversely associated with the PI (*r* = − 0.14; *r* = − 0.18) (Additional file 1: Table S4).

**Table 1:** Profiles of the mother-newborn pairs in this study (*n* = 263)

Variables	Mean ± SD or N (%)
Maternal characteristics	
Age (years)	27.3 ± 4.2
BMI (kg/m^2^)	27.7 ± 3.2
Parity (*n* > 1)	72 (27.4%)
Education status	
Middle school and below	84 (31.9%)
High school	62 (23.6%)
College and above	117 (44.5%)
^a^Economic status	
Below poverty line	61 (23.2%)
Above poverty line	202 (76.8%)
Eating grilled meat (yes)	146 (55.5%)
Passive smoking (yes)	151 (57.4%)
Heating type (self-provided)	65 (24.7%)
Cooking during pregnancy (yes)	117 (44.5%)
*Arterial traffic (< 35 m)	150 (57.0%)
^b^Urinary phenol (μg/mL)	3.7 (1.7, 8.7)
Newborn characteristics	
Gender (female)	118 (44.9%)
Birth weight (BW) (g)	3397.2 ± 429.8
Birth length (BL) (cm)	50.7 ± 1.9
Birth head circumference (BHC) (cm)	34.4 ± 1.4
Gestational age (days)	278.7 ± 7.6
Ponderal index (PI) (g/cm^3^)	2.6 ± 0.3
Cephalization index (CI) (g/cm)	102.7 ± 11.2
^b^Cord blood lead (ng/mL)	24.7 (19.6, 31.2)

Continual variable: Mean ± SD; Categorical variable: N and %

*Arterial traffic refers to the distance between the residence and the main roads

^a^The national standard poverty line is 15,607 yuan/year in Taiyuan city; ^b^Median (P25, P75)

**Table 2:** Urinary creatinine-corrected concentrations of the four PAH metabolites in mothers (*n* = 263 (μg/g Cr))

Exposure metric	P25	P50	P75	GM	Range
2-OH NAP	4.03	6.34	9.41	5.89	0.59–91.02
2-OH FLU	2.23	3.47	5.40	3.34	0.35–23.56
9-OH PHE	1.46	2.88	4.68	2.62	0.35–26.24
1-OH PYR	0.90	1.83	3.03	1.60	0.18–14.42
Ʃ OH PAH	10.40	14.81	22.07	14.82	2.83–104.22

Ʃ OH PAH: the sum of four metabolites of PAH

**Fig. 1:**
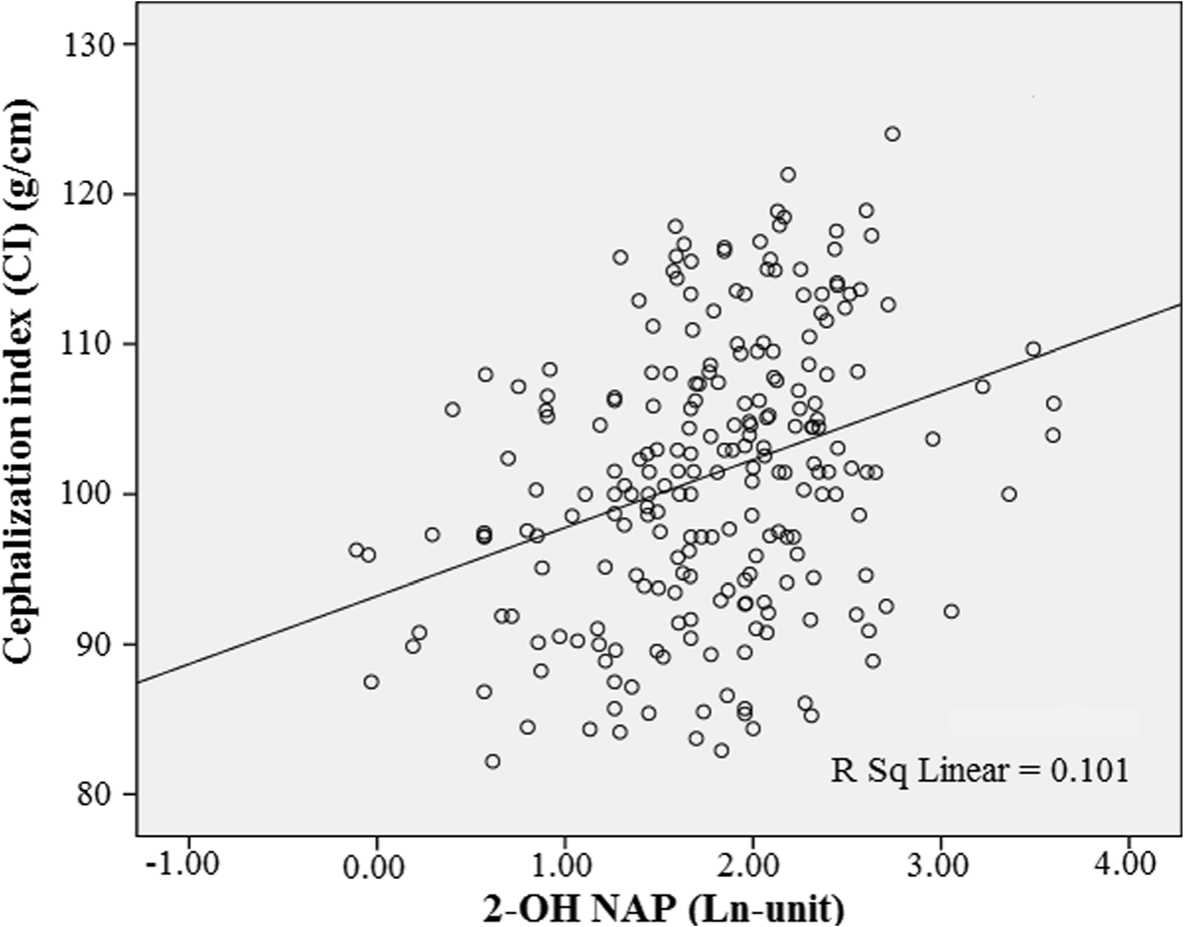
Scatter plots between natural log transformation creatinine-corrected urinary 2-OH NAP (Ln 2-OH NAP) concentration and the cephalization index (CI)

**Fig. 2:**
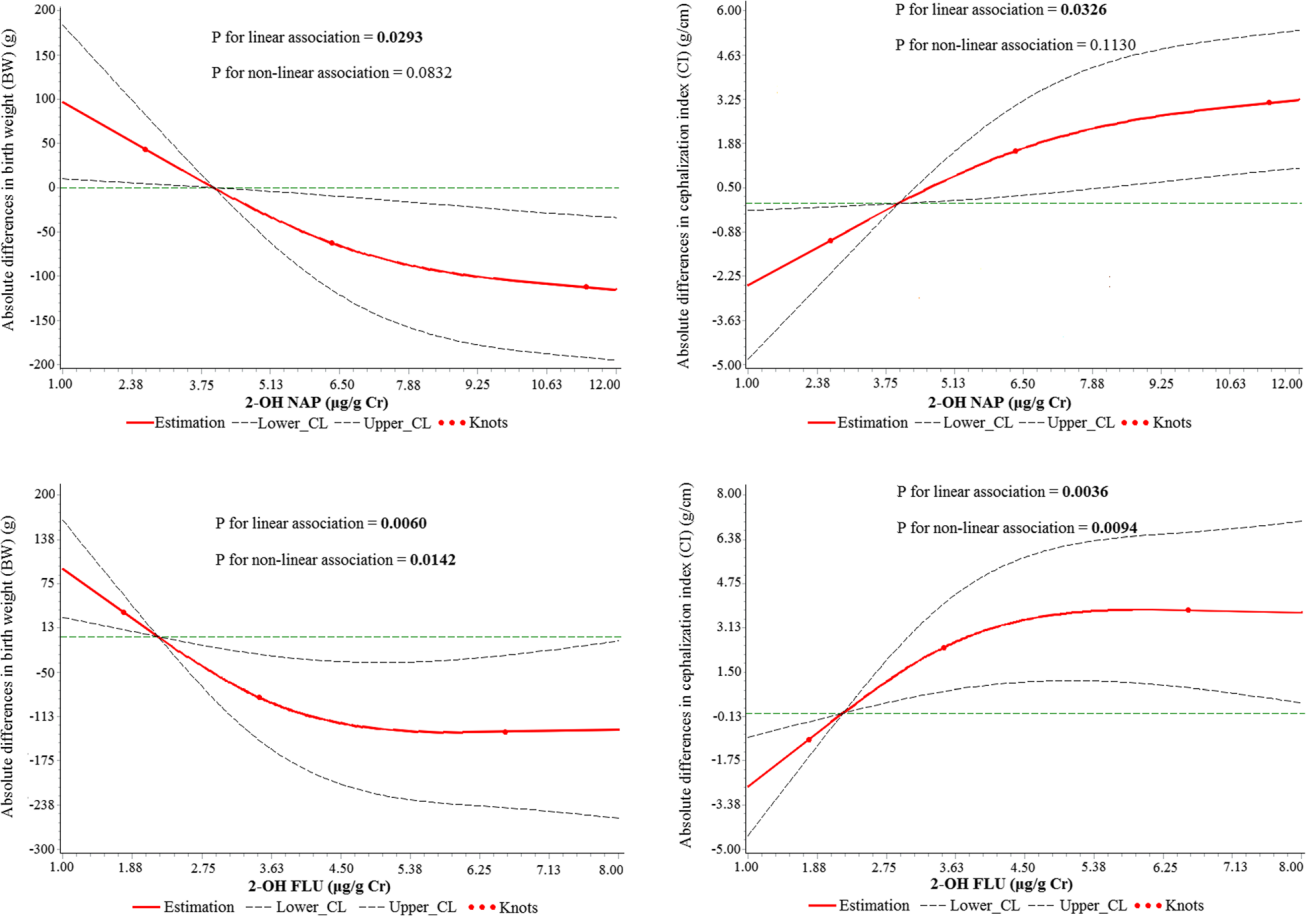
Restricted cubic spline models representing the associations of maternal urinary 2-OH NAP, and 2-OH FLU with birth outcomes (BW and CI) after adjustments for parity, maternal BMI, maternal age, gender of newborn, gestational age, eating grilled meat, passive smoking, and levels of three other PAH metabolites, urinary phenol, and cord blood lead. Dashed lines represent 95% CIs; the red knots represent urinary PAH metabolite concentrations at the 15th, 50th, and 85th percentiles

The scatter plots showed a linear association between 2-OH NAP and the CI, indicating that 10% of the overall variation depends on 2-OH NAP (Fig. 1). According to the detected concentrations of 2-OH NAP, 2-OH FLU, 9-OH PHE, and 1-OH PYR in maternal urine samples, we classified subjects into the low exposure group (the 1st IQR), middle exposure group (the 2nd and 3rd IQR), or high exposure group (the 4th IQR). The adjusted means of birth outcomes across exposure groups are presented in Table 3. General linear model data showed statistically significant differences among the low, middle and high exposure groups with respect to BW and the CI stratified by maternal urinary 2-OH NAP after adjusting for potential confounders. A linear test showed that a higher 2-OH NAP level in maternal urine was associated with a lower newborn BW (p for trend = 0.049) and a higher CI (p for trend = 0.038) after adjusting for parity, maternal BMI, maternal age, gender of newborn, gestational age, eating grilled meat, passive smoking, and 2-OH FLU, 9-OH PHE, 1-OHPYR, urinary phenol, and cord blood lead levels. The 2-OH FLU was also associated with a higher CI (p for trend = 0.027). However, there were no significant trends among the compared levels of other birth outcomes in subjects stratified by urinary levels of 9-OH PHE and 1-OH PYR. Furthermore, the dose-response associations of urinary 2-OH NAP and 2-OH FLU with decreased BW and increased CI were confirmed in the restricted cubic spline models. The results from the general linear model were consistent with the results from restricted cubic spline models (Fig. 2). The *P*-value for linear association was less than 0.05. The different models used to test the association between birth outcomes and urine levels of PAH metabolites are presented in Table 4. We consistently observed that 2-OH NAP was associated with a higher CI in different models. The CI was positively associated with 2-OH NAP (β = 2.09, *p* = 0.035). These data suggested that naphthalene may serve as the primary PAH metabolite associated with the adverse effects of PAH, including decreased BW and increased CI. After eliminating the effect of multicollinearity, Tikhonov regularization also concluded that 2-OH NAP was positively associated with CI (β = 1.80, *p* = 0.035) (Additional file 1: Table S5, Figure S2, and Figure S3). Furthermore, in model 4, 1-OH PYR was negatively associated with PI (β = − 0.09, *p* = 0.004). However, the association of 2-OH FLU and 9-OH PHE with birth outcomes disappeared after adjustments for parity, maternal BMI, maternal age, gender of newborn, gestational age, eating grilled meat, passive smoking, and levels of urinary phenol, cord blood lead, and the other three PAH metabolites. Additionally, maternal BMI and gestational age were correlated with birth outcomes, except for birth length (Table 5). In this study, there were no statistically significant interaction items (Additional file 1: Table S6).

**Table 3:** Birth outcomes of newborns stratified by the four maternal urinary PAH metabolites

Group	N	BW	BL	BHC	PI	CI
2-OH NAP (μg/g Cr)						
Low (< 4.03)	67	3484.7	50.9	34.4	2.6	100.1
Middle (4.03–9.41)	130	3385.1	50.6	34.5	2.6	103.3
High (≥ 9.41)	66	3332.2	50.5	34.3	2.6	104.4
P for trend		**0.049**	0.301	0.525	0.305	**0.038**
2-OH FLU (μg/g Cr)						
Low (< 2.23)	66	3488.4	51.0	34.4	2.6	99.9
Middle (2.23–5.40)	131	3396.0	50.7	34.5	2.6	102.8
High (≥ 5.40)	66	3308.3	50.3	34.4	2.6	105.5
P for trend		0.060	0.118	0.923	0.805	**0.027**
9-OH PHE (μg/g Cr)						
Low (< 1.46)	66	3372.6	50.6	34.5	2.6	103.3
Middle (1.46–4.68)	131	3392.1	50.8	34.4	2.6	102.7
High (≥ 4.68)	66	3431.9	50.5	34.4	2.7	102.3
P for trend		0.567	0.699	0.866	0.404	0.708
1-OH PYR (μg/g Cr)						
Low (< 0.90)	66	3401.6	50.1	34.5	2.7	102.9
Middle (0.90–3.03)	131	3362.2	50.8	34.4	2.6	103.7
High (≥ 3.03)	66	3462.2	50.9	34.4	2.6	100.8
P for trend		0.550	0.108	0.887	0.218	0.439

Adjusted by parity, maternal BMI, maternal age, gender of newborn, gestational age, eating grilled meat, passive smoking, and levels of the other three PAH metabolites, urinary phenol, and cord blood lead. *P*-value < 0.05 was marked in bold

**Table 4:** Associations between four PAH metabolites and birth outcomes by multiple linear regression in different models

Model	Birth outcomes	β (p)			
		2-OH NAP	2-OH FLU	9-OH PHE	1-OH PYR
Model 1	BW	**−73.66 (0.035)**	**−78.83 (0.038)**	−45.04 (0.172)	−46.39 (0.109)
	BL	−0.19 (0.217)	0.06 (0.716)	0.07 (0.636)	0.22 (0.082)
	BHC	− 0.09 (0.401)	− 0.08 (0.503)	− 0.11 (0.312)	− 0.04 (0.675)
	PI	− 0.03 (0.194)	**− 0.08 (0.004)**	**− 0.05 (0.018)**	**− 0.08 (0.001)**
	CI	**2.18 (0.017)**	**2.24 (0.024)**	1.20 (0.164)	1.36 (0.072)
Model 2	BW	**−83.14 (0.009)**	**−82.82 (0.016)**	−51.20 (0.087)	−45.19 (0.086)
	BL	−0.20 (0.180)	0.06 (0.710)	0.06 (0.681)	0.24 (0.055)
	BHC	−0.12 (0.239)	−0.09 (0.405)	− 0.13 (0.196)	−0.04 (0.681)
	PI	−0.04 (0.120)	**−0.08 (0.002)**	**− 0.06 (0.010)**	**−0.08 (0.001)**
	CI	**2.38 (0.005)**	**2.31 (0.012)**	1.34 (0.095)	1.32 (0.061)
Model 3	BW	−71.18 (0.055)	−79.54 (0.125)	28.16 (0.584)	6.27 (0.885)
	BL	−0.32 (0.069)	− 0.20 (0.401)	−0.15 (0.540)	0.55 (0.070)
	BHC	−0.08 (0.506)	−0.05 (0.771)	− 0.16 (0.349)	0.13 (0.372)
	PI	−0.01 (0.858)	−0.02 (0.531)	0.04 (0.294)	**−0.09 (0.005)**
	CI	**2.06 (0.037)**	2.18 (0.115)	−1.06 (0.440)	0.06 (0.960)
Model 4	BW	−71.83 (0.053)	−89.69 (0.088)	38.95 (0.457)	2.54 (0.953)
	BL	−0.31 (0.071)	−0.21 (0.399)	− 0.14 (0.567)	0.55 (0.080)
	BHC	−0.08 (0.506)	− 0.03 (0.858)	− 0.18 (0.296)	0.13 (0.353)
	PI	−0.01 (0.831)	−0.03 (0.402)	0.05 (0.211)	**−0.09 (0.004)**
	CI	**2.09 (0.035)**	2.55 (0.069)	−1.46 (0.297)	0.19 (0.869)

Model 1: unadjusted for covariates

Model 2: adjusted for maternal BMI and gestational age

Model 3: adjusted for potential confounding factors (parity, maternal BMI, maternal age, gender of newborn, gestational age, eating grilled meat, passive smoking, and levels of the other three PAH metabolites)

Model 4: fully adjusted for potential confounding factors (parity, maternal BMI, maternal age, gender of newborn, gestational age, eating grilled meat, passive smoking, and levels of the other three PAH metabolites, urinary phenol, and cord blood lead)

The bold presented a *p*-value less than 0.05

**Table 5:** Associations between all covariates and birth outcomes by multiple linear regression

Variables	BW	BL	BHC	PI	CI
Maternal age					
β	−2.43	−0.07	0.01	0.01	0.09
p	0.695	**0.026**	0.588	0.058	0.593
Maternal BMI					
β	27.52	0.04	0.07	0.02	−0.62
p	**0.001**	0.293	**0.005**	**0.006**	**0.002**
Parity					
β	−48.49	0.20	0.12	−0.07	1.80
p	0.393	0.452	0.509	0.096	0.235
Gestational age					
β	20.84	0.06	0.07	0.01	−0.50
P	**0.001**	**0.001**	**0.001**	**0.001**	**0.001**
Gender (female vs. male)					
β	−67.66	0.07	−0.30	−0.06	1.13
p	0.166	0.751	0.058	0.074	0.386
Passive smoking					
β	−41.20	−0.19	−0.17	0.01	0.14
p	0.402	0.423	0.285	0.921	0.913
2-OH NAP					
β	−71.83	−0.31	−0.08	− 0.01	2.09
p	0.053	0.071	0.506	0.831	**0.035**
Eating grilled meat					
β	8.69	0.19	−0.10	−0.02	−0.57
p	0.859	0.400	0.513	0.524	0.664
Urinary phenol					
β	8.48	0.11	−0.08	−0.01	−0.41
p	0.721	0.310	0.315	0.442	0.519
Cord blood lead					
β	77.93	−0.10	−0.07	0.09	−2.70
p	0.240	0.750	0.759	0.071	0.127

Adjusted for parity, maternal BMI, maternal age, gender of newborn, gestational age, eating grilled meat, passive smoking, and levels of the other three PAH metabolites, urinary phenol, and cord blood lead. The bold text indicates a *p*-value less than 0.05

According to the recorded status of passive smoking (yes or no), the distance from residence to arterial traffic (< 35 m or ≥ 35 m), heating system (collective or self-provided) and cooking experience during pregnancy (yes or no), we classified subjects into two groups and compared the levels of the four PAH metabolites between the groups. As shown in Table 6, we found that the concentrations of 2-OH NAP and 1-OH PYR in maternal urine samples differed between the two groups when stratified by the distance from their residence to arterial traffic. The group of subjects that resided less than 35 m from arterial traffic had a higher level of 2-OH NAP and 1-OH PYR compared to the group that lived more than 35 m away (8.56 μg/g Cr vs. 6.53 μg/g Cr and 2.53 μg/g Cr vs. 2.10 μg/g Cr). The concentration of 1-OH PYR was higher in the group with self-provided heating than in the group with collective heating (2.52 μg/g Cr vs. 2.21 μg/g Cr). We speculated that traffic pollution may contribute to the increased 2-OH NAP concentration of maternal urinary PAH metabolites. Therefore, multiple linear regression analysis was performed for subjects who lived more and less than 35 m away from arterial traffic (Table 7). When the analysis was performed for the lower distance group (< 35 m), an association of 2-OH NAP with low BW and high CI was shown after adjusting for parity, maternal BMI, maternal age, gender of newborn, gestational age, eating grilled meat, passive smoking, and levels of urinary phenol, cord blood lead, 2-OH FLU, 9-OH PHE, and 1-OH PYR. However, we did not reveal a similar association for subjects who lived more than 35 m away from arterial traffic. It is well known that automobile exhaust gases contain high levels of PAH, especially naphthalene. The present data indicated that traffic pollution may be one of the major exposure sources of naphthalene for the general population in Taiyuan city.

**Table 6:** Concentrations of PAH metabolites stratified by possible sources of PAH (μg/g Cr)

Variables	2-OH NAP	2-OH FLU	9-OH PHE	1-OH PYR
Passive smoking				
Yes	7.38 (6.10, 8.67)	4.14 (3.59, 4.69)	3.36 (2.84, 3.87)	2.02 (1.73, 2.32)
No	7.24 (6.39, 8.08)	4.20 (3.70, 4.70)	3.63 (3.15, 4.11)	2.44 (2.10, 2.77)
p	0.770	0.679	0.792	0.435
Distance from arterial traffic				
< 35 m	8.56 (7.05,10.06)	4.44 (3.68, 5.21)	4.01 (3.29, 4.74)	2.53 (2.07, 2.99)
≥ 35 m	6.53 (5.94, 7.12)	4.04 (3.66, 4.42)	3.24 (2.87, 3.61)	2.10 (1.82, 2.37)
p	**0.041**	0.768	0.095	**0.035**
Heating type				
Collective	7.54 (6.71, 8.38)	4.07 (3.61, 4.53)	3.51 (3.07, 3.96)	2.21 (1.91, 2.51)
Self-provided	6.68 (5.46, 7.90)	4.51 (3.89, 5.14)	3.60 (3.05, 4.14)	2.52 (2.15, 2.90)
P	0.239	0.086	0.366	**0.019**
Cooking during pregnant period				
Yes	7.54 (6.37, 8.71)	3.86 (3.39, 4.33)	3.25 (2.82, 3.68)	2.08 (1.73, 2.42)
No	7.25 (6.38, 8.11)	4.48 (3.91, 5.06)	3.79 (3.23, 4.35)	2.46 (2.12, 2.81)
P	0.526	0.275	0.499	0.222

The table presents the mean and 95% confidence interval. The bold presented a *p*-value less than 0.05

**Table 7:** Multiple linear regression model testing associations of 2-OH NAP with birth outcomes in subsets

	BW	BL	BHC	PI	CI
Arterial traffic ≥35 m (*n* = 113)					
2-OH NAP					
β	−6.85	−0.07	−0.16	0.01	−0.07
p	0.904	0.785	0.361	0.934	0.963
Arterial traffic < 35 m (*n* = 150)					
2-OH NAP					
β	−112.56	− 0.46	0.01	− 0.12	3.50
p	**0.035**	0.064	0.999	0.753	**0.016**

Adjusted for parity, maternal BMI, maternal age, gender of newborn, gestational age, eating grilled meat, passive smoking, and levels of the other three PAH metabolites, urinary phenol, and cord blood lead; the bold text indicates *p* < 0.05

## Discussion

We demonstrated for the first time that naphthalene had an adverse effect on birth outcomes. The results showed a significant association of 2-OH NAP level with low BW and high CI. In the present study, we evaluated this dose-response association between 2-OH NAP and birth outcomes after adjusting for levels of 2-OH FLU, 9-OH PHE, and 1-OH PYR and other potential confounders. Further, multiple linear regressions were performed to test the relationship between 2-OH NAP and birth outcomes in different models. There was a consistent positive association between 2-OH NAP and the CI. According to the possible sources of naphthalene, linear regression analysis was performed to test the association in different subsets. The results indicated that one of the major sources of naphthalene may be automobile exhaust in Taiyuan city. A previous study showed that, aside from industrial sources, traffic pollution is the second largest source of naphthalene [22]. The ratio between the traffic source and nonindustrial coal-burning source of naphthalene is 0.77, which indicates that naphthalene is mainly released from traffic pollution, while the other three PAHs are mainly generated by nonindustrial coal-burning. Similar studies have not observed an association of prenatal 2-OH NAP with birth outcomes. The discrepancy between studies may be due to different naphthalene exposure levels. The median concentration of urinary 2-OH NAP (6.34 μg/g) in the present study was higher than that observed in the study by Lamichhane et al. (9.96 ng/g) [18] and Herbstman et al. (3.11 μg/g) [36]. Jing JQ et al. [22] showed that the highest emission load among 16 PAHs was naphthalene in Taiyuan, with a 39.18% contribution rate. The PAH levels from the emission load were consistent with maternal urinary PAH metabolite levels. Researchers have reported in human studies that naphthalene has adverse effects on the neurological function of adults, causing confusion, altered sensorium, listlessness and lethargy, and vertigo [37–40]. Considering the risk of brain damage in newborns, some researchers also advocate banning the use of mothballs on a national level [41]. The CI can serve as a predictive index of neurodevelopment and intelligence quotient in the perinatal period [31]. The present study concluded that naphthalene has an adverse effect on CI, which was in accordance with the biological plausibility theory. We also found that 1-OH PYR was inversely associated with birth outcomes, which was consistent with a previous study [17]. We did not detect a similar association with 2-OH FLU or 9-OH PHE, which presented at a lower concentration in urine than 2-OH NAP.

The biological mechanism of naphthalene-associated adverse effects on birth outcomes remains unclear. Reviewing the related studies, we found that the possible mechanisms included the following aspects. First, naphthalene can destroy the red blood cell and induce fetus hemolytic anemia, especially when the fetus is deficient in glucose-6-phosphate dehydrogenase [2]. Lower hemoglobin can affect the supply of energy and nutrients, which are essential for normal newborn growth and development. Second, the developmental toxicity of naphthalene may contribute to its toxicity to early blastocysts. Accordingly, experimental evidence shows that naphthalene co-cultured with aroclor-induced rat hepatic S-9 fractions exhibited concentration-dependent embryo toxicity in early mouse blastocysts [42]. Third, reactive oxygen species (ROS) formation during naphthalene metabolism can influence newborn development. Naphthalene exposure can produce dose-dependent decreases in cellular glutathione (GSH), adenosine triphosphate (ATP) and cell viability in rat, mouse and human hepatocytes in vitro [43]. Decreased GSH, ATP and cell viability may be suggested as contributing mechanisms in naphthalene-induced cytotoxicity in human exposed to naphthalene. Finally, DNA methylation may be one of the potential mechanisms involved in naphthalene-induced adverse effects on birth outcomes. Several studies have demonstrated that exposure to PAHs can change the methylation of specific genes and global genomic DNA methylation [44–46]. Previous studies have also revealed that the presence of maternal 2-OH NAP is associated with lower methylation levels within the Alu and LINE-1 [47].

We recognize the following limitations in this study. First, there is no external exposure information, which can reflect the level of naphthalene in the environment. Due to the characteristics of naphthalene and complicated detection methods, we did not measure naphthalene concentrations in air. However, PAH metabolites are a validated internal exposure biomarker, and the level of 2-OH NAP can reveal the actual level of naphthalene in the body [48]. Second, biological samples were collected at a single time point (the third trimester), which does not reveal the exposure during the entire pregnancy. Furthermore, the half-life estimate ranged from 2.5–6.1 h for PAH metabolites [49]. We must admit that collecting a single spot urine sample is a flaw of our design. Single spot urine sample measurement may not reflect the effects of long-term exposure to PAH, given the short half-life of PAH metabolites (several hours to days). Thus, multiple urine sample measurements should be used in future studies to evaluate the individual long-term exposure to environmental PAH. Accumulative epidemiologic studies also use single-point urinary PAH metabolites as internal exposure biomarkers, and random single-point PAH metabolites can be used to evaluate PAH exposure levels [47, 50]. Third, none of the birth outcomes considered the population standard for expected growth, which was mainly because the features of recruited subjects were not suitable for considering the clinical relevance in our study. The majority of newborn birth outcomes were in the normal range (98.5%), and there were only 4 low-birth-weight infants and premature infants (1.5%). If we use the population standard as a cutoff point, the models will be unstable and unrealized. Accumulative studies show that low BW (not clinically significant) still increases the risk of suffering from disease in adulthood [13, 51]. The purpose of this study was to investigate the relationship between PAH metabolites and birth outcomes. Our conclusion may be useful for environmental policy making. Finally, the sample size was relatively small in this study. Power analysis showed that the power of this sample size was 0.7 (BW) and 0.8 (CI) after adjusting for covariates (Additional file 1: Figure S4 and Figure S5). For BW analysis, when the 2-OH NAP level increased, BW was significantly decreased in different models after adjusting for potential confounders. This result provided some clues about the effect of naphthalene on BW. For the CI, the power of sample size was enough, and the conclusion that 2-OH NAP was adversely related to a high CI was reliable. Some variables, e.g., passive smoking and the distance from the residence to the arterial road, were solely obtained from self-reported questionnaires. An internal exposure marker, such as cotinine in saliva, can provide accurate information about passive smoking. The geoinformatics analysis can obtain more accurate distance information. However, we do not believe that there is any reason for the study participants to consistently over- or underestimate the passive smoking and distance information. There may be random errors that are against our study hypothesis. These limitations are offset by the comprehensiveness of the analyses, which include the use of multiple models and proper adjustment for potential confounding variables.

## Conclusion

In summary, the present data documents that naphthalene exposure is associated with adverse effects on BW and CI. Therefore, naphthalene exposure from traffic and fossil fuel burning should be decreased during pregnancy to improve the health of newborns.

## Additional file

Additional file 1: Additional file of maternal urinary 2-hydroxynaphthalene and birth outcomes in Taiyuan, China. (DOC 182 kb)

## Abbreviations

1-OH PYR: 1-hydroxypyrene
2-OH FLU: 2-hydroxyfluorene
2-OH NAP: 2-hydroxynaphthalene
9-OH PHE: 9-hydroxyphenanthrene
BHC: Birth head circumference
BL: Birth length
BMI: Body mass index
BW: Birth weight
CI: Cephalization index
HPLC-FLD: High performance liquid chromatography with fluorescence detector
IQR: Median and interquartile range
LOD: Limit of detection
PAH: Polycyclic aromatic hydrocarbon
PI: Ponderal index
Ʃ OH PAH: The sum of four metabolites of PAH
SD: Standard deviation
