# Supplementary material for: Maternal urinary 2-hydroxynaphthalene and birth outcomes in Taiyuan, China
Source: Environ Health. 2018 Dec 20;17:91. doi: 10.1186/s12940-018-0436-4 (PMC6302466; doi:10.1186/s12940-018-0436-4)
Supplement: Supplementary file 1 — Additional file of maternal urinary 2-hydroxynaphthalene and birth outcomes in Taiyuan, China. (DOC 182 kb) [file 12940_2018_436_MOESM1_ESM.doc]

**Supplementary material**

Table S1. Collinearity diagnose statistics PAH metabolites

|  | **Collinearity Statistics** | |
| --- | --- | --- |
|  | **Tolerance ^a^** | **VIF ^b^** |
| 2-OH NAP | 0.726 | 1.378 |
| 2-OH FLU | 0.436 | 2.294 |
| 9-OH PHE | 0.331 | 3.022 |
| 1-OH PYR | 0.362 | 2.759 |

^a^ : more than 0.1 indicated there was weak collinearity among variables

^b^ : less than 10 indicated there was weak collinearity among variables

VIF: variance inflation factor

Table S2. Durbin-Watson value of birth outcomes in regression model

| **Birth outcomes** | **Durbin-Watson ^c^** |
| --- | --- |
| BW | 1.801 |
| BL | 1.756 |
| BHC | 1.741 |
| PI | 1.854 |
| CI | 1.805 |

^c^: the value of approximating to 2 indicated residual error independence

| **PAHs** | **Industrial coking** | **Thermo and industrial burning** | **Non-industrial Coal- burning** | **Traffic** | **Indoor non-coal burning** | **Straw burning for indoor** | **Other source ^b^** | **Total** | **Contribution rate (%)** |
| --- | --- | --- | --- | --- | --- | --- | --- | --- | --- |
| NAP | 37.49 | 27.47 | 25.53 | 19.6 | 18.27 | 1.32 | 0.42 | 130.1 | 39.18 |
| FLU | 5.45 | 0.57 | 6.97 | 0.23 | 1.44 | 0.15 | 0.03 | 14.84 | 4.47 |
| PHE | 18.04 | 1.02 | 18.38 | 0.89 | 5.75 | 0.71 | 0.71 | 45.5 | 13.70 |
| PYR | 6.61 | 0.43 | 4.11 | 0.31 | 1.64 | 0.35 | 0.25 | 13.7 | 4.13 |
| Other 12 PAHs ^c^ | 32.79 | 9.18 | 62.05 | 1.77 | 19.08 | 1.68 | 1.4 | 127.95 | 38.53 |
| Total | 100.38 | 38.67 | 117.04 | 22.8 | 46.18 | 4.21 | 2.81 | 332.09 | 100.00 |
| Contribution rate (%) | 30.23 | 11.64 | 35.24 | 6.87 | 13.91 | 1.27 | 0.85 | 100.00 |  |

Table S3. Sources list 16 PAHs emissions in Taiyuan (2010) ^a^ (t)

^a^: Jing JQ, Yu LY, Xin HX, Bin LU, Shu T, Rong W: Estimation of annual emission and distribution characteristics of polycyclic aromatic hydrocarbons(PAHs) in Taiyuan. China Environmental Science 2013, 33(1):14-20.

^b^: Non-traffic source, electrolytic aluminium, hill fire, straw burning for outdoor, and oil refining

^c^: ACY, ACE, ANT, FLA, BaA, CHR, BbF, BkF, BaP, IcdP, DahA, and BghiP

Table S4. Spearman correlation matrix between the numerous exposure metrics and birth outcomes

|  | **2-OH NAP** | **2-OH FLU** | **9-OH PHE** | **1-OH PYR** | **BW** | **BL** | **BHC** | **PI** | **CI** |
| --- | --- | --- | --- | --- | --- | --- | --- | --- | --- |
| 2-OH NAP | 1.00 | 0.42^**^ | 0.47^**^ | 0.42^**^ | -0.15^*^ | -0.02 | -0.03 | -0.13^*^ | 0.17^**^ |
| 2-OH FLU |  | 1.00 | 0.71^**^ | 0.68^**^ | -0.15^*^ | 0.04 | 0.02 | -0.18^**^ | 0.15^*^ |
| 9-OH PHE |  |  | 1.00 | 0.76^**^ | -0.08 | 0.05 | 0.02 | -0.14^*^ | 0.08 |
| 1-OH PYR |  |  |  | 1.00 | -0.09 | 0.09 | 0.03 | -0.18^**^ | 0.09 |
| BW |  |  |  |  | 1.00 | 0.56^**^ | 0.62^**^ | 0.57^**^ | -0.96^**^ |
| BL |  |  |  |  |  | 1.00 | 0.49^**^ | -0.27^**^ | -0.51^**^ |
| BHC |  |  |  |  |  |  | 1.00 | 0.23^**^ | -0.42^**^ |
| PI |  |  |  |  |  |  |  | 1.00 | -0.58^**^ |
| CI |  |  |  |  |  |  |  |  | 1.00 |

^*^ :< 0.05; ^**^ :< 0.01

Table S5. Associations between four PAH metabolites and birth outcomes by Tikhonov regularization after adjusted for confounders (Ridge k=0.1)

|  | **β (p)** |  |  |  |  |
| --- | --- | --- | --- | --- | --- |
|  | **BW** | **BL** | **BHC** | **PI** | **CI** |
| 2-OH NAP | -62.18 (0.052) | -0.28 (0.058) | -0.08 (0.465) | -0.01 (0.889) | **1.80 (0.035)** |
| 2-OH FLU | -63.61 (0.112) | -0.13 (0.474) | -0.04 (0.751) | -0.02 (0.404) | 1.75 (0.103) |
| 9-OH PHE | 16.63 (0.651) | -0.08 (0.654) | -0.11 (0.342) | 0.02 (0.451) | -0.66 (0.500) |
| 1-OH PYR | 1.89 (0.952) | 0.41 (0.061) | 0.08 (0.426) | **-0.07 (0.002)** | 0.11 (0.897) |

Bold indicates p-value < 0.05.

Ridge k: the parameter of Tikhonov regularization in ridge tracer goes to stable region

Table S6. The interaction of 2-OH NAP and other three PAH metabolites on birth outcomes

|  | **BW** | **BL** | **BHC** | **PI** | **CI** |
| --- | --- | --- | --- | --- | --- |
| 2-OH NAP |  |  |  |  |  |
| β | -67.4 | 0.4 | -0.1 | -0.1 | **1.2** |
| P | 0.510 | 0.334 | 0.620 | 0.099 | **0.001** |
| 2-OH FLU |  |  |  |  |  |
| β | -115.0 | -0.1 | -0.3 | -0.1 | 2.0 |
| P | 0.202 | 0.839 | 0.239 | 0.243 | 0.396 |
| 9-OH PHE |  |  |  |  |  |
| β | 165.6 | -0.2 | 0.02 | **0.2** | -4.2 |
| P | 0.065 | 0.579 | 0.943 | **0.015** | 0.083 |
| 1-OH PYR |  |  |  |  |  |
| β | -122.6 | 0.4 | -0.2 | **-0.2** | 2.5 |
| P | 0.122 | 0.306 | 0.339 | **0.008** | 0.234 |
| 1. OH NAP *   2-OH FLU |  |  |  |  |  |
| β | 78.4 | -0.7 | 0.4 | 0.1 | 0.5 |
| P | 0.937 | 0.267 | 0.469 | 0.284 | 0.663 |
| 2-OH NAP *  9-OH PHE |  |  |  |  |  |
| β | -246.7 | 0.1 | -0.3 | 0.2 | 5.7 |
| P | 0.093 | 0.902 | 0.490 | 0.051 | 0.133 |
| 1. OH NAP *   1-OH PYR |  |  |  |  |  |
| β | 254.5 | 0.1 | 0.5 | 0.2 | -2.5 |
| P | 0.137 | 0.953 | 0.269 | 0.371 | 0.351 |

Bold indicates p-value < 0.05.

**
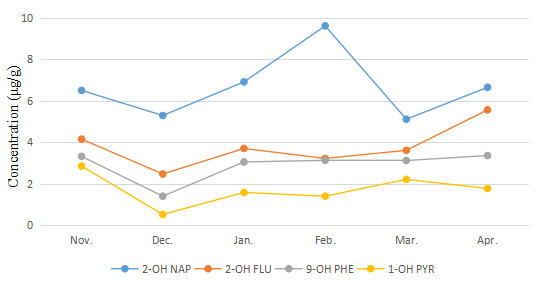
Figure S1. Urinary PAH metabolites level in different months**


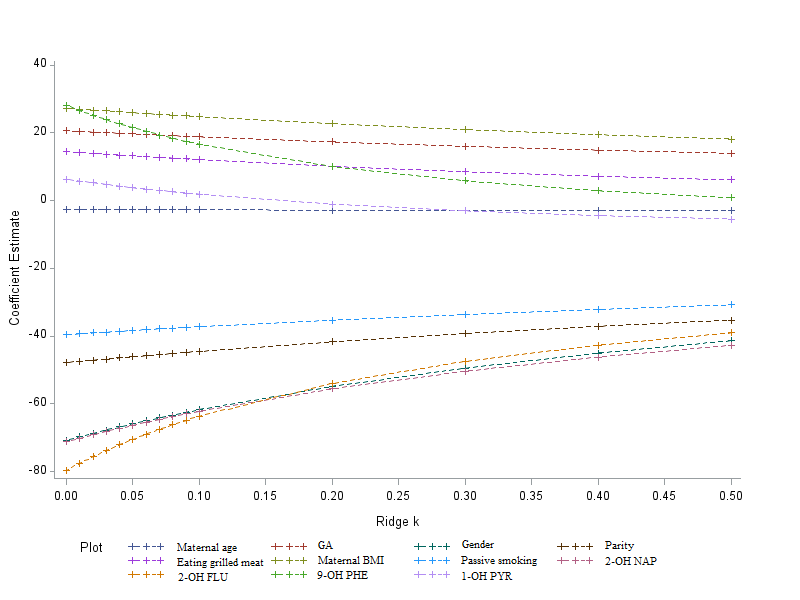


**Figure S2. Ridge tracer of the variables for birth weight (BW)**

**
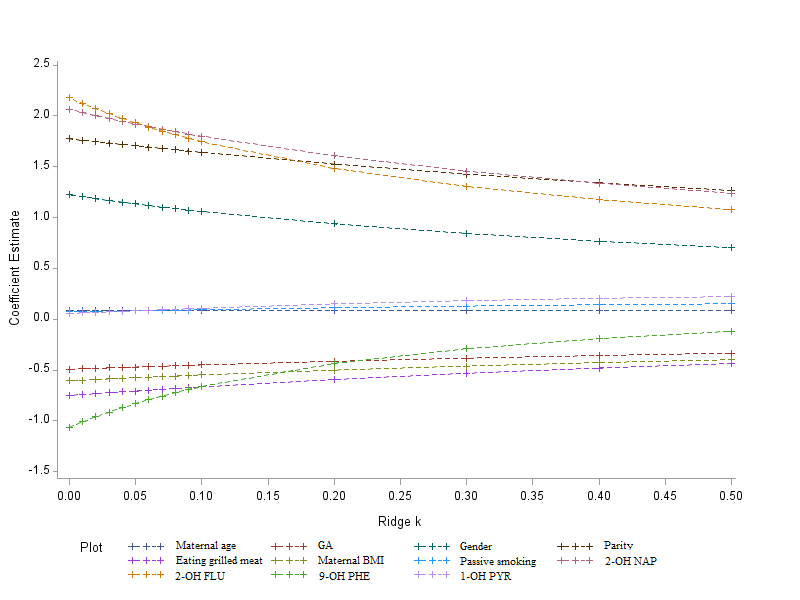
Figure S3. Ridge tracer of the variables for cephalization index (CI)**

**
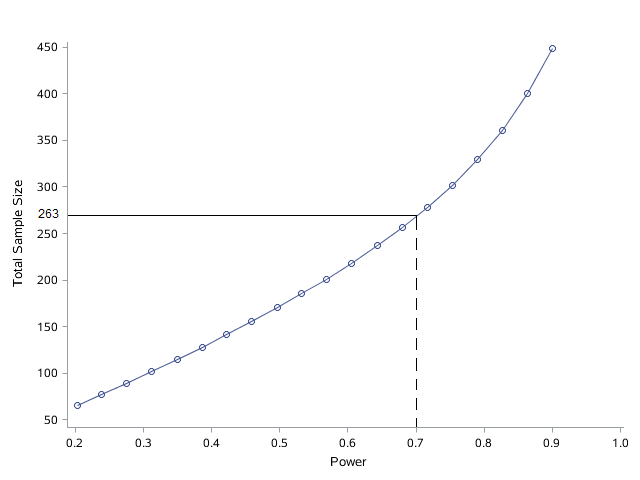
**

**Figure S4. The association between power and sample size (2-OH NAP and BW) (power=0.7)**

**
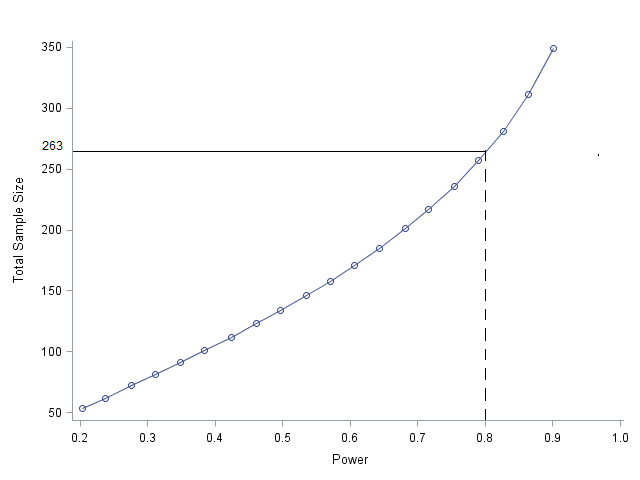
**

**Figure S5. The association between power and sample size (2-OH NAP and CI) (power=0.8)**
